# Exploring the role of non-formal training in an international youth mobility on young adult’s perception of appearance and dental aesthetics

**DOI:** 10.1186/s12903-026-09109-5

**Published:** 2026-07-10

**Authors:** Ayşegül Hazır, Ömer Faruk Sönmez, Begüm Atsü, Saadet Atsü

**Affiliations:** 1 Vocational School of Health Services Prosthetic Dental Technology Program, Kırıkkale University, Kırıkkale, Türkiye; 2 Vocational School of Health Services Oral Health Program, Kırıkkale University, Kırıkkale, Türkiye; 3 Clinical Child Psychology, Anglia Ruskin University, Cambridge, UK; 4 Faculty of Dentistry Department of Prosthetic Dentistry, Kırıkkale University, Kırıkkale, Türkiye

**Keywords:** Dental aesthetics, Body image, Non-formal education, Social media, PIDAQ, SATAQ-4

## Abstract

**Background:**

Dental aesthetic concerns in young adults are shaped by sociocultural appearance pressures and media-derived ideals, yet education-based interventions targeting these upstream determinants have received limited attention in dentistry. This exploratory study examined whether a brief non-formal educational programme delivered within an Erasmus+ youth mobility context was associated with short-term changes in sociocultural appearance attitudes and the psychosocial impact of dental aesthetics.

**Methods:**

A single-group, quasi-experimental pre-test/post-test design was used. Thirty-four young adults (aged 18–30; 23 from Türkiye, 11 from France) completed the Sociocultural Attitudes Toward Appearance Questionnaire-4 (SATAQ-4) and the Psychosocial Impact of Dental Aesthetics Questionnaire (PIDAQ) immediately before (T0) and immediately after (T1) a five-day programme integrating media literacy, body-positivity activities, and oral health education. Pre–post differences were tested using Wilcoxon signed-rank tests with Holm adjustment, with rank-biserial correlations as effect sizes. Internal consistency was estimated using Cronbach’s α and McDonald’s ω. Reporting followed the TREND and TIDieR guidelines.

**Results:**

From T0 to T1, SATAQ-4 total scores decreased (r_rb = − 0.782, 95% CI [− 0.956, − 0.536]) and PIDAQ total scores decreased (r_rb = − 0.655, 95% CI [− 0.882, − 0.345]). All subscales showed changes in the direction anticipated by the study hypothesis, with reductions in negative PIDAQ domains and an increase in Dental Self-Confidence. All comparisons remained significant after Holm adjustment (p_Holm ≤ 0.008). Internal consistency was generally high, except for the SATAQ-4 Thin/Low Body Fat subscale at T1 (α = 0.62).

**Conclusions:**

Participation in the programme was associated with short-term shifts in self-reported appearance attitudes and dental-aesthetic outcomes. Findings should be considered preliminary and hypothesis-generating; the absence of a control group, immediate post-test timing, and use of self-report measures preclude causal inference. Controlled designs with longer-term follow-up are needed.

## Introduction

Prosthetic dental treatment aims to restore oral function and to enhance psychosocial well-being through improvements in dental appearance [1, 2]. Contemporary expectations regarding dental aesthetics are shaped by clinical determinants, sociocultural norms, media-derived ideals, and individual psychological factors [3, 4]. Among young adults, the close link between dental appearance, self-esteem, and perceived social acceptance underscores the importance of subjective aesthetic appraisal in evaluating treatment outcomes [5].

Digital media and social networking platforms have increasingly shaped how individuals construct and evaluate appearance-related standards [6, 7]. Body dissatisfaction refers to the negative subjective evaluation of one’s own physical appearance, arising from a perceived discrepancy between an individual’s actual body and an internalized ideal standard. Frequent exposure to curated and idealized content is linked to heightened body dissatisfaction and pressure to align with narrow beauty norms [8]. How these dynamics extend to dental aesthetics is clinically relevant, as such perceptions may shape patient motivation, expectations, and satisfaction with prosthetic and cosmetic outcomes [9].

Patient-reported outcome measures (PROMs) quantify subjective experience alongside clinical indices [10]. The Psychosocial Impact of Dental Aesthetics Questionnaire (PIDAQ) is a validated instrument for young populations [11]. It assesses four domains, namely dental self-confidence, social impact, psychological impact, and aesthetic concern, and is widely used in orthodontics and prosthetic/aesthetic dentistry [12–14]. Reported internal consistency for the PIDAQ subscales is high (Cronbach’s α ≈ 0.85–0.91) [11, 15].

The Sociocultural Attitudes Toward Appearance Questionnaire-4 (SATAQ-4) measures the internalization of appearance ideals shaped by media, peers, family, and significant others, alongside perceived appearance-related pressures [16, 17]. It builds on the SATAQ-3 by independently assessing pressure sources and distinct ideals such as thinness and muscularity [16]. Reported subscale α values range from 0.87 to 0.95 [16].

Although the SATAQ-4 was originally developed in the context of eating disorder research, its internalization and pressure subscales — including those targeting thinness and muscularity ideals — are theoretically relevant to dental aesthetic perception. Social media platforms promote a unified idealized appearance that conflates bodily, facial, and dental attractiveness; thus, individuals who internalize thinness or fitness ideals from social media may simultaneously adopt unrealistic dental appearance standards. This conceptual overlap justifies the inclusion of the SATAQ-4 alongside the PIDAQ in the present study.

Appearance ideals refer to socially constructed standards of physical attractiveness that individuals internalize as personal benchmarks for evaluating their own appearance, shaped and reinforced through peer norms, family feedback, and digital media content [16]. Body image concerns are associated with dental-aesthetics–related perceptions and psychosocial impacts. Greater body image disturbance is linked to higher dental aesthetic concern and stronger social and psychological impacts. In addition, negative body image attitudes may generalize to a more unfavourable evaluation of one’s dental appearance [18, 19]. Family, peers, and media are the three primary sociocultural pathways shaping body image and satisfaction with dental appearance [16]. In contemporary contexts, social media arguably exerts the most pervasive influence. Filters and photo-editing applications have normalized digitally altered smiles as aspirational standards, and such images are increasingly presented in clinical settings as desired treatment outcomes [20]. Social media content promoting idealized body weight, muscularity, and facial symmetry frequently presents dental appearance as an integrated component of overall attractiveness, making it difficult for viewers to disentangle dental ideals from broader bodily ones. The “Hollywood Smile” is typically a monochromatic and excessively opaque whiteness that departs from natural tooth morphology. Such ideals may increase willingness to pursue invasive restorative procedures at the expense of intact tooth structure [21].

Prosthetic interventions can improve quality of life but must be planned with attention to biological, functional, and patient-related risks. Biologically, tooth preparation can impose thermal and mechanical stress on the pulp, and restoration margins can compromise periodontal integrity [22, 23]. Functionally, treatment may produce occlusal disturbances, phonation difficulties, and mechanical failures from material fatigue [24]. Patient-related risks include difficulty adapting to an altered dental appearance and dissatisfaction driven by unrealistic or externally imposed aesthetic ideals [25, 26].

Short, intensive educational formats such as youth exchange programmes can change attitudes and behaviours [27, 28]. By combining intercultural learning, peer interaction, and experiential methods, they can strengthen critical awareness of idealized appearance norms [29–31]. The Erasmus+ Programme is one such setting, and its youth exchanges combine personal development goals with intensive experiential learning. Unlike formal education, non-formal education is structured but flexible and voluntary, emphasizing active involvement, reflection, and learning-by-doing [32].

A substantial literature has examined the psychosocial consequences of dental aesthetics [5, 33–35], but largely in clinical contexts comparing pre- and post-treatment outcomes. Education-based interventions integrating psychoeducation with sociocultural determinants of appearance have received limited attention in dentistry. Searches in PubMed, MEDLINE, and Google Scholar combining terms such as “PIDAQ,” “SATAQ-4,” “dental aesthetics,” “body image,” “social media influence,” “non-formal education,” and “experiential learning” identified no studies evaluating non-clinical, education-oriented interventions targeting dental aesthetic perception alongside broader sociocultural appearance processes. This gap is significant. If sociocultural appearance pressures are upstream determinants of dental aesthetic dissatisfaction, then interventions targeting these pressures may offer a more sustainable approach than addressing dental concerns in isolation. This is particularly relevant given the growing influence of social media on appearance ideals among young adults. Media literacy and body-positivity programmes have shown value in helping young people interrogate unrealistic appearance ideals and develop healthier aesthetic attitudes [36], but their relevance to dental aesthetic perception and to motivations for prosthetic treatment remains under-studied.

Conceptually, sociocultural appearance pressures may influence dental aesthetic concerns through internalization of ideals and appearance-based social comparison, with social media amplifying these processes. Yet no study to date has examined whether a brief psychoeducational intervention can simultaneously address both. The present study directly targets this gap. If brief psychoeducational programmes can reduce internalization and perceived pressure, they may also attenuate dental-aesthetics–related psychosocial burden and support more realistic expectations for aesthetic dental treatment. The aim of this study was to examine whether participation in a five-day non-formal educational programme, delivered within an international Erasmus+ youth mobility context, was associated with short-term changes in sociocultural attitudes toward appearance and in the psychosocial impact of dental aesthetics among young adults aged 18 to 30. A pre-test/post-test design with the SATAQ-4 and PIDAQ was used. The null hypothesis (H0) is that the intervention will not produce statistically significant differences between pre- and post-test scores on the SATAQ-4 or the PIDAQ.

## Method

### Participants and data collection

Ethical approval for the study was obtained from the Kırıkkale University Non-Interventional Research Ethics Committee (Decision No: 2026.02.27; Date: 26.02.2026). A single-group, quasi-experimental pre-test and post-test design was used to examine changes associated with participation in an Erasmus+ KA152-YOU youth exchange program delivered during the Türkiye mobility component hosted in Kırıkkale.

The intervention consisted of a structured non-formal educational program integrating media literacy training, a guided social media–based activity on beauty ideals, body-positivity–oriented sessions, and oral and dental health workshops. Written informed consent was obtained from all participants prior to data collection. To protect confidentiality, each participant was assigned a unique alphanumeric study code, and no directly identifying information was retained in the analytic dataset.

Data were collected at two time points using the same assessment battery. Baseline data (T0) were obtained immediately before the start of program activities. Post-intervention data (T1) were collected on the final day of the program, immediately after completion of the educational activities.

The study population comprised young adults aged 18 to 30 years who were officially selected to participate in the Erasmus+ KA152-YOU youth exchange program in Türkiye. After selection, participants were eligible if they met the age criterion, provided written informed consent, and completed both the baseline and post-intervention assessments. Participants were excluded if they declined consent or withdrew from the study, had missing data at either measurement occasion, or reported severe psychological distress requiring referral to urgent mental health services in accordance with participant safety procedures.

### Participant flow and recruitment

**Fig. 1:**
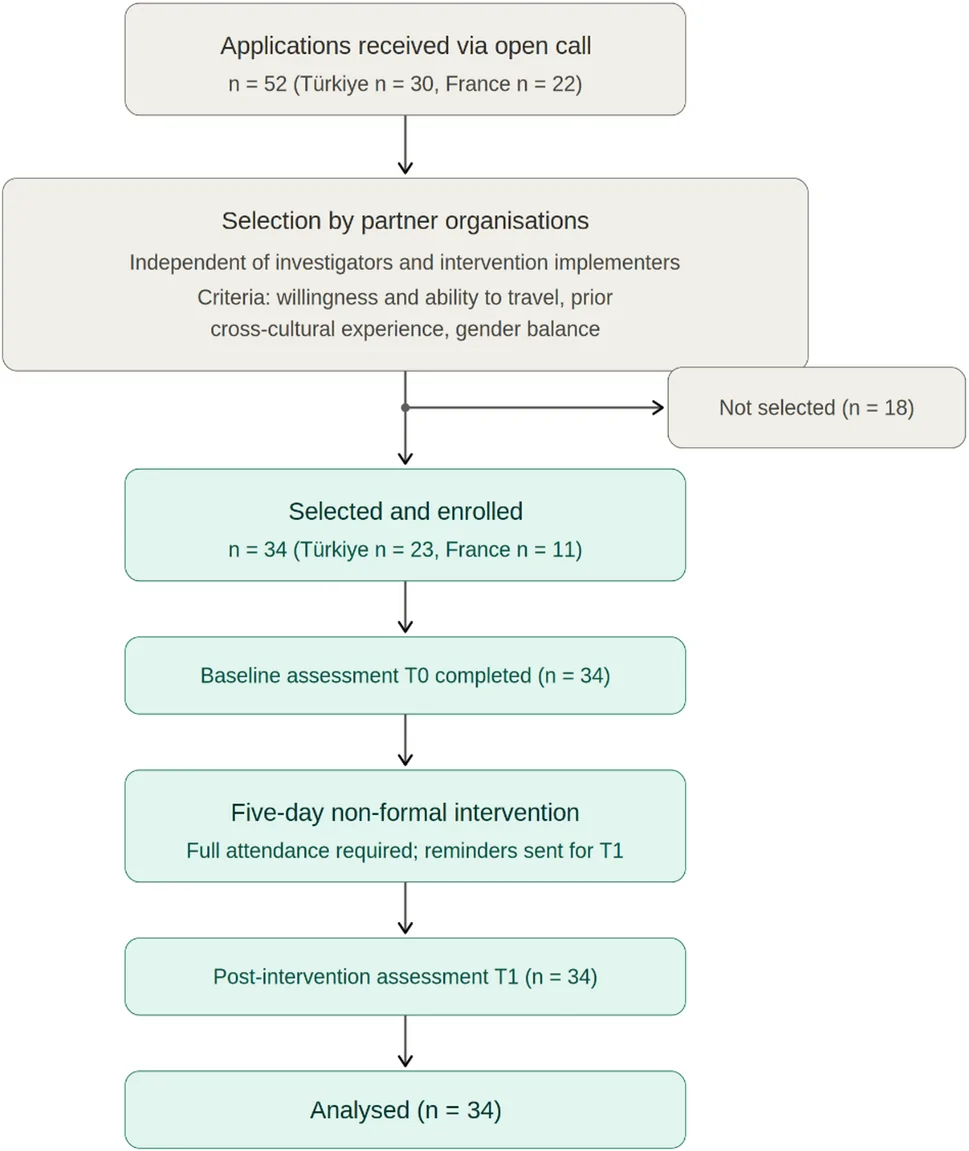
Participant flow diagram

Participants were recruited through an open call disseminated via the websites and communication channels of the partner organisations in Türkiye and France. A total of 52 applications were received (*n* = 30 from Türkiye and *n* = 22 from France). Selection was conducted by the partner organisations independently of the investigators and intervention implementers, on the basis of applicants’ willingness and ability to travel, English language competence, prior experience in cross-cultural contexts, and gender balance, in accordance with Erasmus+ KA152-YOU programme rules. Selection criteria did not include prior knowledge of, interest in, or expressed motivation regarding beauty standards, body image, or dental aesthetics. The partner organisations conducting the selection were not informed that pre- and post-test assessments would be administered or that the exchange would be reported as a research study, so applicants were neither selected for nor against responsiveness to the research outcomes. From the 52 applications, 34 applicants were selected for participation (*n* = 23 from Türkiye, *n* = 11 from France); the remaining 18 applicants were not selected. All 34 selected participants attended the programme, completed the baseline assessment (T0) on the first day, and completed the post-intervention assessment (T1) on the final day. There were no exclusions due to missing data and no attrition between T0 and T1, although completion of the post-intervention assessment by a small number of participants required follow-up reminder emails from the research team. Full participation was a requirement for receiving the Youthpass certificate of completion, and this was communicated to applicants through the open call. A participant flow diagram is provided in Fig. 1.

The overall scope of the youth exchange was designed by the project team at the Kırıkkale University Faculty of Dentistry at the application stage of the Erasmus+ KA152-YOU project. The detailed session design, intervention delivery, and outcome evaluation were carried out by the research and intervention team based at the Vocational School of Health Services, which included a psychologist and a social worker. The partner organisation in France was responsible for participant recruitment and logistics on the French side. As described in the Participant Flow paragraph, applicant selection was conducted by the partner organisations independently of the investigators and intervention implementers.

### Sample size

The a priori sample size calculation was performed prior to data collection using the pwr package in R statistical software, based on the paired-samples t-test framework appropriate for pre-test/post-test designs with repeated measures on the same individuals. Assuming a medium effect size (Cohen’s dz = 0.50), a two-tailed significance level of α = 0.05, and 80% statistical power, the minimum required sample size was determined to be *n* = 34. This calculation was conducted in advance and submitted as part of the ethics application to the Kırıkkale University Non-Interventional Research Ethics Committee. Because the standard Erasmus+ KA152-YOU programme allocation provided for 25 participants, additional eligible young adults meeting the inclusion criteria were recruited alongside the funded participants in order to reach the calculated minimum, yielding the final analytic sample of *n* = 34. The additional participants completed the same intervention activities and assessments as the funded participants and were not differentiated in the analysis.

### Intervention

The intervention comprised a structured, non-formal educational program delivered over five consecutive days during the youth exchange. Session design and facilitation followed the principles outlined in the Council of Europe’s *Manual for Facilitators* to support group engagement, reflective learning, and active participation [37]. The program was grounded in Kolb’s Experiential Learning Theory and combined interactive activities targeting appearance ideals, body image, media literacy, and awareness of dental aesthetics [38]. The overall structure was intentionally intensive, with daily modules that integrated experiential tasks, guided reflection, and group discussion. The agenda of the intervention and session plans are available in Table 3 of the Supplementary Material.

The program included four core components. First, the initial day focused on group dynamics and the establishment of a supportive learning climate. Activities were used to promote psychological safety and to enable participants to discuss potentially sensitive topics, such as body image and appearance-related concerns, in an open and respectful environment. Second, media literacy sessions were delivered on two separate days and were designed to strengthen critical appraisal of appearance-related media messages. These sessions used applied case examples of digital manipulation and guided exercises to examine how commercial and social media content may shape appearance ideals. Reflective writing and role-based activities were incorporated to support cognitive reappraisal and to encourage more compassionate self-evaluations. A full decomposition of the intervention by component domain, approximate dose, and conceptually targeted construct is provided in Supplementary Table 6, alongside the TIDieR and TREND checklist (Supplementary Tables 4 and 5) and the session-by-session agenda (Supplementary Table 3); component-level inference is not attempted in the present design, as discussed in the Limitations.

Third, modules on dental aesthetics and oral health were delivered on two days and were facilitated by a dentist. These sessions aimed to contextualize dental appearance within biological and functional realities, emphasizing individual variation, natural aesthetics, and oral hygiene. Interactive workshops addressed basic principles relevant to smile aesthetics and encouraged participants to distinguish clinically appropriate goals from externally imposed or homogenized ideals. Fourth, cross-cultural learning was embedded throughout the program via structured peer interaction and end-of-day reflections. Environmental considerations related to consumption and aesthetic practices were also integrated, and participants documented key learning points in personal journals to reinforce consolidation of learning.

Baseline assessment (T0) was administered after informed consent and before any program activities. Post-intervention assessment (T1) was completed on the final day of the program using the same instruments. Data were collected electronically using an online survey platform. Participants completed a demographic questionnaire and two validated self-report measures: the Sociocultural Attitudes Toward Appearance Questionnaire-4 (SATAQ-4) and the Psychosocial Impact of Dental Aesthetics Questionnaire (PIDAQ).

Both questionnaires are previously validated instruments published in the literature. The PIDAQ was developed by Klages et al. [11] and the SATAQ-4 was developed by Schaefer et al. [16]; neither instrument was developed for this study.

The demographic form captured participant characteristics including age, gender, and country of residence. The PIDAQ comprises 23 items across four subscales: Dental Self-Confidence, Social Impact, Psychological Impact, and Aesthetic Concern. Items are rated on a five-point Likert scale ranging from 0 (“not at all”) to 4 (“very strongly”), where higher Dental Self-Confidence scores indicate greater confidence and higher scores on the remaining subscales indicate greater negative psychosocial impact. The SATAQ-4 assesses internalization of appearance ideals and perceived sociocultural pressures from key sources (family, peers, and media), as well as appearance-based comparison tendencies. Items are rated on a five-point Likert scale from 1 (“strongly disagree”) to 5 (“strongly agree”), with higher scores indicating greater internalization and perceived pressure.

This study was reported in accordance with the Transparent Reporting of Evaluations with Nonrandomized Designs (TREND) statement, with the intervention description further structured according to the Template for Intervention Description and Replication (TIDieR) checklist [39, 40]. Within this framework of compliance, the theoretical basis of the intervention, the educational materials used, the step-by-step content of the procedures applied, and the practitioners’ expertise levels are detailed in Table 4 in Supplementary Material. The intervention rationale, materials, facilitator roles, and procedural elements were specified to enable replication in comparable settings.

### Data analysis

All analyses were conducted using the R statistical computing environment (R Foundation for Statistical Computing). Participant responses were screened for completeness and eligibility. Only participants who provided data at both the baseline assessment (before the program) and the post-intervention assessment (immediately after the program) were included in paired analyses.

All study materials, including the consent form, demographic questionnaire, PIDAQ, and SATAQ-4, were administered in their original validated English versions. English was the working language of the Erasmus+ KA152-YOU exchange, and adequate English proficiency was a prerequisite for selection, confirmed by the partner organisations. No translation or cross-cultural adaptation was therefore undertaken.

The Psychosocial Impact of Dental Aesthetics Questionnaire and the Sociocultural Attitudes Toward Appearance Questionnaire-4 were scored according to their established subscale structures. For each participant, subscale scores were computed as the arithmetic mean of the relevant item responses so that subscale values remained on the original response scale. For the Psychosocial Impact of Dental Aesthetics Questionnaire, the dental self-confidence dimension is positively keyed (higher values indicate greater dental self-confidence). In contrast, higher values on the aesthetic, psychological, and social impact dimensions indicate greater negative psychosocial impact. For total score reporting, a single direction of interpretation was used: positively keyed items were reverse-scored prior to creating the total score, so that higher total scores reflect greater negative psychosocial impact of dental aesthetics, while the positively keyed subscale was reported in its original direction for interpretability. For the Sociocultural Attitudes Toward Appearance Questionnaire-4, higher values indicate stronger internalization of appearance ideals and greater perceived sociocultural pressures.

Participant characteristics were summarized using counts and percentages for categorical variables and using the mean and standard deviation, as well as the median, interquartile range, minimum, and maximum for continuous variables. Outcome scores were summarized at baseline and post-intervention using the mean and standard deviation, and the within-participant change was summarized as the mean and standard deviation of the paired difference (post-intervention minus baseline).

The primary objective was to test whether outcome scores changed from baseline to post-intervention within the same participants. For each total score and each subscale score, distributional assumptions for parametric paired testing were evaluated by examining the distribution of within-participant difference scores and by applying the Shapiro–Wilk test for normality. Because the difference distributions deviated from normality, paired comparisons were performed using the Wilcoxon signed-rank test. All tests were two-sided, and statistical significance was evaluated at an overall significance level of 0.05.

Because multiple outcomes were tested (two total scores and multiple subscale scores), the familywise error rate was controlled using the Holm adjustment. Both unadjusted and Holm-adjusted probability values were reported.

For each Wilcoxon signed-rank test, the rank-biserial correlation was reported as the effect size, with 95% confidence intervals computed using a Fisher transformation. The rank-biserial quantifies the degree to which post-intervention scores are lower or higher than baseline scores: negative values indicate that post-intervention scores are generally lower than baseline, and positive values indicate the opposite.

Internal consistency reliability was evaluated separately at baseline and post-intervention for each subscale using two complementary indices: Cronbach’s alpha and McDonald’s omega total. These estimates were reported to describe the degree of internal coherence of each scale at each measurement occasion and to support the interpretation of change scores.

In addition to the primary pre–post comparisons, several exploratory analyses were conducted and are reported in Supplementary Material. For all participants, Sociocultural Attitudes Toward Appearance Questionnaire-4 total and Psychosocial Impact of Dental Aesthetics Questionnaire total scores were summarized at baseline and post-intervention, and the within-participant mean change was calculated as the post-intervention score minus the baseline score for each total outcome. Associations between post-intervention total scores were evaluated using Spearman’s rank correlation. Additional exploratory correlation analyses examined (i) the association between baseline total scores, (ii) the association between total change scores of the two instruments, and (iii) the association between baseline total scores and total change scores for each instrument. These associations were visualized using scatterplots with fitted trend lines and confidence bands. Finally, exploratory subgroup comparisons assessed whether total change scores differed by field of study (dentistry versus non-dentistry) and by country of residence (France versus Türkiye) using the Wilcoxon rank-sum test for independent samples; groups were described using the mean and standard deviation of the change scores. As these analyses were exploratory and not part of the primary outcomes, they were presented in the supplementary material without being used to draw confirmatory inferences.

## Results

Thirty-four participants completed both assessments (T0 and T1). The sample was young (mean age 22.59 years, SD 3.07; range 18–29). Most participants were female (65%). Two-thirds resided in Türkiye (68%) and one-third in France (32%). The largest academic subgroup was Dentistry (35%), with the remainder distributed across several disciplines (Table 1).

**Table 1:** Baseline characteristics of participants (T0)

Characteristic	Value (mean ± SD; median (IQR); range, or *n* (%).)
Sample size	*N* = 34
Age (years)	22.59 ± 3.07; median 22.00 (IQR 21.00–24.00); range 18.00–29.00
Sex	
Female	22 (65%)
Male	12 (35%)
Country of residence	
France	11 (32%)
Türkiye	23 (68%)
Level of study	
Associate degree student	1 (2.9%)
Bachelor’s graduate	6 (18%)
Bachelor’s student	19 (56%)
High school graduate	2 (5.9%)
Master’s graduate	3 (8.8%)
Master’s student	3 (8.8%)
Field of study	
Business	3 (8.8%)
Communication	1 (2.9%)
Dentistry	12 (35%)
Education	2 (5.9%)
Engineering/IT	2 (5.9%)
Health sciences	2 (5.9%)
Language	5 (15%)
Law	2 (5.9%)
Medicine	1 (2.9%)
Not reported / missing	3 (8.8%)
Psychology	1 (2.9%)

Across the full sample, both primary outcomes improved from baseline (T0) to post-intervention (T1). The negative psychosocial impact of dental aesthetics decreased, reflected by a lower PIDAQ total score at T1 than T0, and sociocultural appearance pressures/internalisation also declined, reflected by a lower SATAQ-4 total score at T1 than T0 (Table 2). The same directional pattern was observed at the subscale level: all SATAQ-4 subscales decreased over time, whereas within PIDAQ, the negatively keyed domains (Aesthetic Concern, Psychological Impact, Social Impact) decreased, while Dental Self-Confidence increased (Table 2).

**Table 2:** PIDAQ and SATAQ-4 subscale scores at baseline (T0) and post-intervention (T1) and difference

Measure	T0 mean (SD).	T1 mean (SD).	Difference (T1 − T0) mean (SD).
Total scores			
PIDAQ total	1.75 (1.18)	0.73 (0.79)	−1.03 (1.47)
SATAQ-4 total	2.81 (1.17)	1.82 (0.61)	−1.00 (1.37)
PIDAQ subscales			
Aesthetic Concern (AC)	1.66 (1.47)	0.51 (0.83)	−1.15 (1.68)
Dental Self-Confidence (DSC)*	2.05 (1.22)	3.17 (0.93)	+ 1.12 (1.46)
Psychological Impact (PI)	1.89 (1.20)	0.84 (0.89)	−1.05 (1.58)
Social Impact (SI)	1.54 (1.34)	0.65 (0.95)	−0.89 (1.64)
SATAQ-4 subscales			
Internalization: Muscular/Athletic	3.06 (1.19)	1.99 (0.70)	−1.06 (1.34)
Internalization: Thin/Low Body Fat	2.71 (1.02)	1.90 (0.59)	−0.81 (1.06)
Perceived pressures: Family	2.82 (1.39)	1.74 (1.01)	−1.08 (1.71)
Perceived pressures: Media	3.00 (1.46)	2.02 (1.18)	−0.98 (1.69)
Perceived pressures: Peers	2.44 (1.49)	1.35 (0.63)	−1.09 (1.63)

*The PIDAQ Dental Self-Confidence (DSC) subscale is positively keyed

All total and subscale scores changed from T0 to T1, with all comparisons remaining significant after Holm adjustment (all p_Holm ≤ 0.008; Table 3). Both total scores improved. The SATAQ-4 total decreased with a large effect and a strong negative rank-biserial correlation (r_rb = − 0.782, 95% CI [− 0.956, − 0.536]), indicating post-test values were generally lower than baseline. The PIDAQ total also decreased (r_rb = − 0.655, 95% CI [− 0.882, − 0.345]) (Table 3).

**Table 3:** Within-participant pre–post effects for SATAQ-4 and PIDAQ total and subscale scores (Wilcoxon signed-rank)

Outcome	V	*p*-value (raw)	p_Holm	Rank-biserial (r_rb)	r_rb (95% CI)
Total scores					
SATAQ-4 total	57.5	< 0.001 (0.000112)	0.001 (0.00112)	−0.782	[− 0.956, − 0.536]
PIDAQ total*	91	0.001 (0.00121)	0.006 (0.00607)	−0.655	[− 0.882, − 0.345]
SATAQ-4 subscales					
Internalization: Thin/Low Body Fat	38.0	< 0.001 (0.000169)	0.002 (0.00152)	−0.813	[− 0.960, − 0.568]
Internalization: Muscular/Athletic	33	< 0.001 (0.0000651)	< 0.001 (0.000716)	−0.848	[− 0.990, − 0.624]
Perceived pressures: Family	68.5	0.002 (0.00210)	0.008 (0.00839)	−0.663	[− 0.905, − 0.333]
Perceived pressures: Peers	26	< 0.001 (0.000623)	0.004 (0.00436)	−0.812	[− 1.000, − 0.536]
Perceived pressures: Media	42.0	0.003 (0.00341)	0.008 (0.00839)	−0.696	[− 0.935, − 0.337]
PIDAQ subscales					
Dental Self-Confidence (DSC)*	461.5	< 0.001 (0.000220)	0.002 (0.00176)	+ 0.748	[0.481, 0.931]
Social Impact (SI)	100	0.006 (0.00637)	0.008 (0.00839)	−0.570	[− 0.849, − 0.214]
Psychological Impact (PI)	77.5	0.002 (0.00246)	0.008 (0.00839)	−0.644	[− 0.880, − 0.315]
Aesthetic Concern (AC)	39.5	< 0.001 (0.000921)	0.006 (0.00553)	−0.757	[− 0.949, − 0.459]

Paired comparisons were performed using the Wilcoxon signed-rank test. r_rb is the rank-biserial correlation; its sign reflects the direction of change, with magnitudes around 0.1, 0.3, and 0.5 commonly interpreted as small, medium, and large

Within SATAQ-4, the largest shifts were in internalization: Muscular/Athletic (r_rb = − 0.848) and Thin/Low Body Fat (r_rb = − 0.813). Perceived pressures also decreased Peers and Media showed large-to-moderate effects (*r* ≈ 0.71 and 0.61), and Family somewhat smaller but still meaningful all r_rb confidence intervals were entirely below zero (Table 3).

Within PIDAQ, negatively keyed domains decreased including Aesthetic Concern, Psychological Impact, and Social Impact while Dental Self-Confidence increased (r_rb = + 0.748, 95% CI [0.481, 0.931]), with confidence intervals supporting a consistent direction of change (Table 3).

Internal consistency was generally high for PIDAQ subscales at both time points and remained high for most SATAQ-4 subscales; however, the Internalization: Thin/Low Body Fat subscale showed comparatively lower reliability at post-test (Table 4), which should be considered when interpreting change in that specific domain.

**Table 4:** Internal consistency at baseline (T0) and post-intervention (T1)

Scale / Subscale	α		ω	
PIDAQ	(T0)	(T1)	(T0)	(T1)
Aesthetic Concern (AC)	0.933	0.922	0.936	0.935
Dental Self-Confidence (DSC)	0.951	0.967	0.953	0.969
Psychological Impact (PI)	0.912	0.872	0.916	0.882
Social Impact (SI)	0.958	0.953	0.959	0.955
SATAQ-4				
Internalization: Thin/Low Body Fat	0.785	0.615	0.884	0.686
Internalization: Muscular/Athletic	0.919	0.841	0.921	0.851
Perceived pressures: Family	0.922	0.920	0.923	0.925
Perceived pressures: Peers	0.979	0.915	0.980	0.917
Perceived pressures: Media	0.966	0.926	0.968	0.928

α = Cronbach’s alpha; ω = McDonald’s omega total

The following analyses were prespecified as exploratory and are reported separately so that they remain distinct from the confirmatory pre–post comparisons above. They are presented for descriptive purposes only and are not used to draw confirmatory inferences. Full details and figures are provided in the Supplementary Material.

Exploratory correlation analyses described positive associations between SATAQ-4 and PIDAQ total scores at baseline (T0) and post-intervention (T1), and a positive correspondence between pre–post change scores across the two measures (Supplementary Figs. 1–5). Exploratory subgroup comparisons of change scores did not show statistically significant differences by study field (dentistry vs. non-dentistry) or by country (France vs. Türkiye) for either PIDAQ total change or SATAQ-4 total change (all *p* > 0.32; Supplementary Tables 1 and 2). These comparisons were underpowered due to small subgroup sizes and should not be interpreted as evidence of equivalence.

In the dataset folder through the data repository, total score means and means in difference were also reported for each participant.

## Discussion

This single-group quasi-experimental study describes pre–post changes observed in young adults who participated in a brief non-formal educational programme delivered within an international youth mobility context. From baseline to post-intervention, SATAQ-4 total and subscale scores declined, PIDAQ domains reflecting negative psychosocial impact decreased, and Dental Self-Confidence increased. These patterns are compatible with the theoretical premise that appearance pressures and internalization are upstream determinants of appearance-related distress. The findings are best interpreted as preliminary and hypothesis-generating: participation in the programme coincided with shifts in self-reported outcomes rather than demonstrating that the programme produced these shifts.

Because the study used a single-group, uncontrolled design, a common set of structural limitations applies to all of the interpretations that follow and is therefore stated once here rather than repeated in each paragraph. The absence of a control group, the immediate post-test timing, the reliance on self-report measures, and the modest sample size together preclude causal inference and limit precision for subgroup and mechanistic analyses. Accordingly, the associations and shifts discussed below are presented as patterns compatible with the study’s conceptual premise rather than as effects demonstrably produced by the programme, and these constraints are examined in greater detail in the Limitations section.

### Concordance between sociocultural attitudes and dental-aesthetic outcomes

SATAQ-4 and PIDAQ totals were positively associated at both time points, consistent with the view that dental aesthetic concerns are embedded within broader body image and appearance-evaluation processes rather than representing an isolated oral-health domain. This pattern is compatible with the Tripartite Influence Model, in which family, peer, and media influences shape appearance outcomes through internalization of ideals and appearance-based social comparison [41]. Within this framework, sociocultural pressures from family, peers, and media are proposed to influence the internalization of appearance ideals, which in turn shapes appearance-related perceptions (including dental-aesthetic perceptions) and downstream psychosocial impact. We use this model as an interpretive lens for the observed concordance, not as a mechanism tested by the present data.

In exploratory analyses, change scores on the two instruments were positively correlated, indicating that participants who reported larger shifts in sociocultural appearance attitudes also tended to report larger shifts in dental-aesthetic psychosocial outcomes. This correspondence is descriptive: change-score correlations are susceptible to shared method variance (both instruments are self-report Likert measures completed at the same two time points), measurement error, and regression-to-the-mean, particularly given the underpowered subgroup analyses.

### Pre–post reductions in sociocultural appearance internalization

The largest changes in SATAQ-4 scores were observed in the internalization domains. This is theoretically relevant because internalization is often conceptualized as a proximal cognitive process linking exposure to appearance ideals with appearance-related dissatisfaction. The observed pattern is broadly consistent with literature on school-based and social media literacy programmes, which has reported favourable changes in critical appraisal skills and body image–related outcomes [42–44].

Several alternative explanations remain plausible. Without a comparison condition, decreases in SATAQ-4 scores may reflect repeated-measurement effects, short-term changes in mood or salience related to the programme environment, demand characteristics, or social desirability in a group setting that explicitly encouraged critical reflection on appearance ideals. The timing of the post-test may also have influenced responses, as data were collected at the end of the programme, when participants may have experienced emotional intensity, fatigue, group cohesion, or a desire to report positively. In addition, participants with higher baseline endorsement tended to show larger decreases, which may be compatible with meaningful short-term change but is also consistent with regression-to-the-mean. Finally, the lower post-test internal consistency of the SATAQ-4 Thin/Low Body Fat subscale suggests that findings for this specific domain should be interpreted with additional caution.

### Pre–post changes in dental-aesthetic psychosocial outcomes

PIDAQ scores showed short-term decreases in domains reflecting dental-aesthetic psychosocial burden: Aesthetic Concern, Psychological Impact, and Social Impact decreased, while Dental Self-Confidence increased. As with the SATAQ-4 outcomes, these are self-reported measures collected in a context that may favour positive reporting.

The increase in Dental Self-Confidence is of particular interest because it suggests that body-positivity and media-literacy content may, at least conceptually, alter the cognitive frame through which participants evaluate their own dental appearance. The data do not allow separation of whether this reflects a targeted shift in dental aesthetic beliefs or a broader improvement in self-evaluation that generalised across appearance-related domains. The result is compatible with prior evidence that broader body image concerns relate to dental appearance perceptions [18] and tentatively extends that literature by suggesting that interventions targeting general appearance attitudes may also affect oral appearance domains.

### Possible explanatory pathways

The programme incorporated experiential learning, media literacy, body-positivity activities, oral health education, and cross-cultural group reflection. These elements may offer plausible explanatory pathways for the observed short-term changes, but the present design does not allow the active components to be identified. Experiential learning principles may have facilitated participant engagement with appearance-related topics more than a purely didactic format would have [38]. Cognitive dissonance theory also offers a possible explanatory framework, as some prevention programmes use critical evaluation of idealized appearance norms to reduce endorsement of those norms [45]. In the present study, however, such mechanisms remain hypothetical.

The cross-cultural setting should also be viewed as a contextual feature rather than as an empirically tested moderator. Bringing together participants from Türkiye and France may have created opportunities to discuss appearance norms across different cultural frames. This is relevant because standards of attractiveness and smile aesthetics are socially and culturally shaped, and cultural differences in preferred tooth colour, tooth display, and smile characteristics have been reported [46]. However, the study was not designed or powered to test cultural differences. The exploratory subgroup analyses by country were underpowered, and the absence of statistically significant differences should not be interpreted as evidence of equivalence.

### Hypothesis-generating implications for prosthetic dentistry

Any extension of these findings to prosthetic dental practice must be cautious. The sample comprised young adults voluntarily participating in an educational exchange rather than patients seeking aesthetic or prosthetic treatment, and only a subset reported a dentistry background. The study therefore does not provide direct evidence about patients in clinical decision-making contexts. With that constraint in mind, the findings raise a hypothesis worth testing in clinical samples: that brief psychoeducational content aimed at improving media literacy and reducing rigid internalization of idealized appearance standards may have a role as an adjunct to clinical consultation. If supported in patient populations, such content could in principle be delivered as a short pre-treatment module, a structured chairside script, or a digital decision aid that helps patients differentiate between digitally enhanced images and biologically plausible outcomes [20, 21]. The aim of testing this would not be to discourage aesthetic treatment, but to explore whether it supports shared decision-making, calibrates expectations, and reduces the likelihood of pursuing invasive procedures to match externally imposed ideals [22–26]. Whether PROMs such as the PIDAQ are useful in this context, as structured aids to identify patients experiencing high psychosocial burden related to dental appearance rather than as screening tools for treatment eligibility, is a separate empirical question that the present non-clinical sample cannot address.

### Limitations

Several limitations should be considered when interpreting these findings. First, the study employed a single-group pre-test and post-test design without a comparison condition. As a result, causal attribution to the intervention is limited, and observed changes may partially reflect threats to internal validity such as maturation, contextual influences related to participation in an international mobility program, or repeated-measurement effects. Controlled designs, ideally randomized, are required to determine whether the intervention produces improvements beyond what would be expected from time, novelty, or assessment reactivity alone.

Second, the timing of the post-intervention assessment introduces two related concerns. Post-test data were collected on the final day of the program, at the moment of participant departure. This context is characterized by group closure, emotional intensity, fatigue, and intensified social bonding, any of which may bias self-reports in a positive direction independent of genuine attitudinal change and may have artificially inflated the apparent intervention effect. Beyond this immediate response-bias concern, the durability of any genuine change also remains unknown, since short-term shifts in attitudes and self-reported psychosocial outcomes may attenuate once participants return to their usual social environments and social media exposure. Future work should administer the post-test at a more emotionally neutral point, separated from departure activities, and include follow-up assessments at meaningful intervals such as 3, 6, and 12 months to evaluate maintenance of change and the need for booster sessions.

Third, although the sample size was adequate to detect medium within-participant effects, it provided limited precision for subgroup analyses and moderator testing. The absence of statistically significant differences by country or field of study should therefore be interpreted cautiously, as the study was not powered to detect small-to-moderate subgroup effects. Larger, multi-site samples would enable more robust examination of demographic, cultural, and educational moderators, as well as assessment of heterogeneity of treatment response. Although the absence of attrition between T0 and T1 is a methodological strength, it was supported by the certificate-of-completion requirement and, for a small number of participants, by reminder emails from the research team to complete the post-intervention assessment; both factors may have generated mild compliance pressure alongside the response-bias considerations already discussed.

Finally, participation was based on voluntary application and selection into an Erasmus+ program. This introduces potential selection bias, as participants may differ from the general young adult population in motivation, openness to reflective learning, and baseline attitudes toward appearance and health. Consequently, generalizability to broader community or clinical samples is uncertain, and is particularly limited in relation to patients seeking aesthetic dental treatment, who differ systematically from this educational sample. Replication in more diverse settings, including non-selected community samples and clinical populations seeking aesthetic dental treatment, would strengthen external validity.

## Conclusion

The findings of this study should be considered preliminary. In a single-group pre–post design without a comparator, participation in a five-day non-formal educational youth exchange programme was associated with short-term decreases in internalization scores and more favourable dental-aesthetics–related self-reported scores among young adults. These results are compatible with the conceptual premise that appearance-related sociocultural processes and dental aesthetic perceptions are intertwined and may be amenable to brief, structured psychoeducational approaches. The data generate the hypothesis that integrating media literacy and appearance-related psychoeducation into patient preparation could be worth investigating in prosthetic and aesthetic dentistry. The present non-clinical sample does not establish such applicability. Confirmation will require replication in controlled designs, in more diverse populations, and with longer-term follow-up.

## Data Availability

The datasets used and/or analyzed during the current study are available from the Mendeley data repository using following DOI number: https://doi.org/10.17632/nkv4pnsmy5.1.

## Abbreviations

AC: Aesthetic Concern
CI: Confidence Interval
DSC: Dental Self-Confidence
dz: Cohen’s dz
H0: Null Hypothesis
KA152-YOU: Erasmus + Key Action 152 – Youth
PIDAQ: Psychosocial Impact of Dental Aesthetics Questionnaire
PROMs: Patient-Reported Outcome Measures
pwr: Power analysis package in R
r_rb: Rank-biserial correlation
SATAQ-4: Sociocultural Attitudes Toward Appearance Questionnaire-4
SD: Standard Deviation
SI: Social Impact
T0: Baseline – Pre-test
T1: Post-intervention
TIDieR: Template for Intervention Description and Replication
TREND: Transparent Reporting of Evaluations with Nonrandomized Designs
α: Cronbach’s Alpha
ω: McDonald’s Omega Total
